# Micromechanical Characterisation of Ni/PU Hybrid Foams

**DOI:** 10.3390/ma13173746

**Published:** 2020-08-24

**Authors:** Martin Reis, Kristian König, Stefan Diebels, Anne Jung

**Affiliations:** Applied Mechanics, Saarland University, D-66123 Saarbrücken, Germany; martin.reis@uni-saarland.de (M.R.); s8krkoen@stud.uni-saarland.de (K.K.); s.diebels@mx.uni-saarland.de (S.D.)

**Keywords:** micromechanical testing, experimental mechanics, digital image correlation, open-cell foams, hyrid foams, microtensile, microbending

## Abstract

The computer-aided design of individual parts and the desire for weight reduction and material savings require further development of new hybrid materials. Ni/PU hybrid foams as a new hybrid material offer great potential for the production of components that are lightweight and yet can absorb large amounts of energy. The development of this structured material is at its beginning and mechanical characterisation on all scales is necessary. Experimental investigations on individual struts must be carried out on the micro scale to understand the structure-properties-relationship. Inspite of the challenges raising due to the complex geometry of the struts, tensile tests, three-point bending tests and micro sections are presented in this work. Due to the stiff Ni coating on the outer diameter of the struts, the resistance against bending is around five times as high as against tensile loading. The correlation between the behaviour of the struts and the macroscopic material behaviour validates the planned use of the foams as energy absorbers.

## 1. Introduction

In nature, large cavities in load bearing structures are usually not filled with solid material. Looking at trees, corals and even bones, a complex internal structure is observable. Here, much volume is filled with a fine structure of little material surrounded by fluids. This is made possible by a load-optimised design of the structure. As a generic term such structures are called foams. When talking about foams, a distinction must first be made between open-cell, partially closed-cell and closed-cell foams. Open-cell foams consist of individual struts connected to build pores and thus form a macroscopic foam. The facets between all struts are open and there are no cell walls. In partially closed-cell foams, the cell walls are partially or completely closed for some pores. In closed-cell foams almost every pore is closed and the foam is not permeable. A first technical description of foams can be found by Ashby et al. [1]. Presently, closed-cell foams are often used in sandwich panels, where a foam core is surrounded on two sides by thin metal sheets. These structural elements have a low weight but high stiffness especially against bending, which enables lighter and stiffer constructions of bridges and aircraft as well as the construction of blast resistance for buildings. Due to the low stiffness of open-cell foams, they are mainly used as functional materials in filters or in batteries [2,3]. However, a new approach also shows the possibility of using open-pored hybrid foams as structural elements. The results of Vesenjak et al. [4] and Duarte et al. [5] show promising results for tubes filled with open-pored hybrid foams for use as structural elements. This study focuses on hybrid foams whose struts are coated by an electro-deposition process. Since this process requires a floating of electrolyte through the foam, the coating is only possible for open-cell foams. Therefore, only results for open-cell foams are presented in the following.

The high energy absorption capacity of foams can be explained by their deformation behaviour. Three regimes are visible when looking at the stress-strain curve of a macroscopic foam sample, starting with a linear-(pseudo)elastic regime up to plastic collapse stress (PCS). In PCS, the first pores begin to collapse, causing the deformation of further pores until the entire pore layer is damaged, which initiates the second regime. In the second regime, pore layer by pore layer collapses and forms a stress plateau at high strain. This plateau ends in a strong stress increase with almost no further increase in strain [1,6,7,8]. Based on this description, a clear scale dependence of the deformation behaviour is recognisable. The macroscopic deformation leads to a deformation of the pores on the meso scale. As the pores are compressed, struts on the micro scale are deformed in compression and bending. This relationship between structure and mechanical properties strongly demonstrates the need to understand the behaviour on the micro scale and thus the necessity to test individual struts.

The non-uniform and complex structure of the individual struts complicates experimental testing. The size of the struts and the force to be expected during deformation preclude the use of standard macroscopic testing machines. For these reasons, there is almost no literature available on the mechanical testing of individual struts [9]. Kaya et al. [10,11] have conducted tensile tests on individual struts of an open-cell steel foam. During the tests, the strain is measured using Digital Image Correlation (DIC) and the struts are supported by an especially designed bearing using a frictional connection. The material under investigation are struts extracted from a sintered steel foam. The typical cross-sectional geometry consists of three lens-shaped hollow steel bars connected to each other at the corners and forming a concave triangular cavity in the middle of each strut. The special geometry of the cross-section of these foam struts leads to a special failure observation. The struts tear at the connection points resulting in a crack of the struts. Jung et al. [12] studied the struts of Ni/Al hybrid foams, i.e., nickel (Ni) coated aluminium struts. Furthermore, pure aluminium struts of open-cell Al foams are examined. The mechanical tests are carried out with an especially designed micromechanical testing device [8]. The struts are supported by a form-fit connection by casting with a soldering alloy. As a result, Jung et al. [12] presented an increase in stiffness of the hybrid foam that is not only generated by the pure increase in cross-sectional area of the struts but also strongly enhanced by the hard and stiff Ni coating affecting the tensile and the bending stiffness.

In reality, struts in foams are not often loaded in tension and the bending of struts has a higher relevance to understand macroscopic foam behaviour. Fila et al. [13] presented three-point bending tests on rectangular specimens extracted from the cell walls of a closed-cell aluminium foam. As testing setup, a custom-designed bending device was evolved and calibrated using thin aluminium sheets. Fila et al. [13] were able to show that the material parameters measured by nanoindentation are two to three times higher than the values measured in the bending tests as an effect of the localised measurement by nanoindentation. They and were thus able to substantiate the need for realistic tests on individual cell walls. Since foams are bio-inspired, bone trabeculae are similar to foam struts and the test routine applied to these specimens is similar to the testing of individual struts of a metal foam. Jungmann et al. [14] extracted trabecula from bone and performed three-point bending tests on these samples. DIC is used for the evaluation of local strain fields. It shows large positive strains on the opposite side of the mandrel and negative strains at the point of load application. In Reis et al. [15] three-point bending tests on individual struts are used to prove the accuracy of the DIC setup, without presenting the experimental results of the strut tests in detail. Two-point bending tests on cell walls or on individual struts are not common in the literature.

This paper presents experiments on individual struts of a nickel-polyurethane (Ni/PU) hybrid foam. This material is a composite foam, which combines the low cost of an open-cell PU foam with the high stiffness of a nanocrystalline Ni coating [16]. The results of the study are presented in four parts. First, an optical analysis is performed to measure the thickness of the coating and the size of the PU core for five struts. Second, microtensile tests are described and executed with an especially designed clamping device to ensure good alignment of the struts and high quality DIC measurements. Third, three-point microbending tests are presented in which a new evaluation routine is used to calculate the bending stiffness for each of the struts. Finally, the results of tensile and bending tests are compared, showing a geometrical influence on the results of the tests.

## 2. Materials and Methods

### 2.1. Ni/Pu Hybrid Foams and Strut Preparation

Ni/PU hybrid foams with a pore size of 20 ppi (pores per inch) were produced according to the electrodeposition process described in previous work [16,17,18]. For both microtensile and three-point microbending experiments, individual struts must be carefully extracted from the macroscopic foam without causing pre-deformation of the struts. For this purpose, a fine side cutter is used and smaller areas of about ten pores are first cut out of the macroscopic foams. In the second step unnecessary struts are carefully removed one by one until a one-strut sample is obtained.

**Figure 1:**
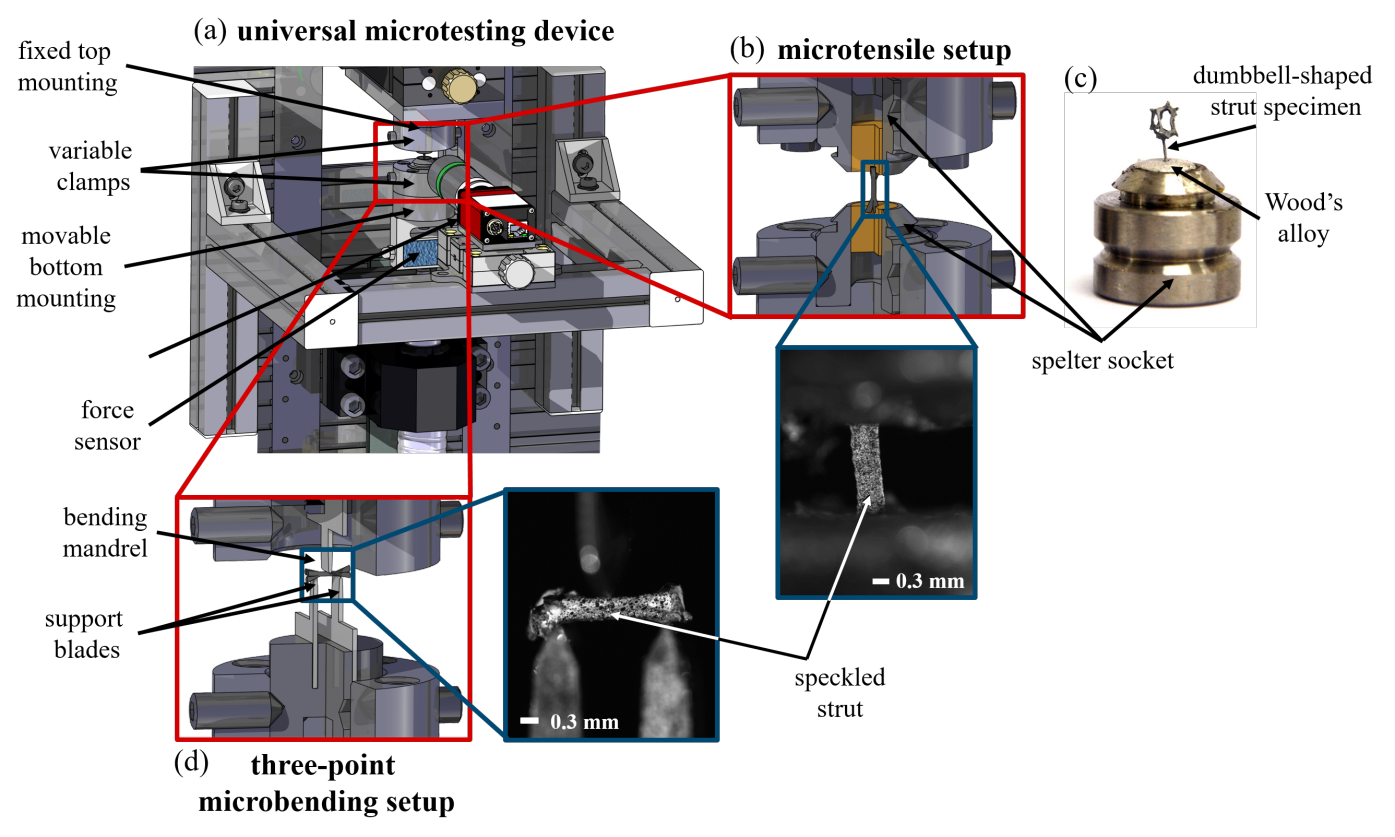
Experimental setup for micromechanical experiments: (**a**) detail of the universal micromechanical testing device including the different modules for (**b**) microtensile testing and (**d**) three-point microbending experiments on individual struts; (**c**) shows the dumbbell-shaped strut specimen for tensile testing already moulded into the spelter socket on one side by Wood’s alloy.

Similar to macroscopic experiments, different sample geometries must be used for the two types of microexperiments. While for the three-point microbending tests only entire struts are placed on two fine support blades acting as bearings near the adjacent nodes, for the microtensile tests stable force application points have to be applied. Therefore, an additional pore remains on each strut at both the lower and upper node, creating a kind of dumbbell-shaped specimen (see Figure 1c) for the tensile tests. These remaining pores are used for fixation of the strut to the testing device.

### 2.2. Universal Micromechanical Testing Device

The small dimensions combined with the complex and varying geometry of each strut reject the use of a standard testing device. Hence, a special custom-build testing rig is designed (see Figure 1). The construction uses a stepper motor combined with a high precision drive resulting in a step resolution of 0.2 μm for a load up to 1000 N. Here, the force is measured by different s-bracket force sensors (20 N, 100 N) from the KD40s series of ME-Messsyteme, Henningsdorf, Germany with a measurement resolution of 0.1%. For displacement and strain measurements the setup allows the attachment of one or more cameras to perform 2D and 3D DIC evaluations using the commercial software Istra4D, Dantec Dynamics, Ulm, Germany. Furthermore, the device is characterised by its great modularity. Different clamping options allow tensile (Figure 1b) and compression experiments on single pores and single struts, as well as two-point and three-point bending experiments (Figure 1d) on individual struts [8].

For the microtensile experiments, the fixation of the single struts in the universal microtesting device is realised by moulding the dumbbell-shaped strut specimen with the two pores into a spelter socket at the top and the bottom mounting of the testing device using Wood’s alloy to guarantee for a stable but easy removable fixation. While the moulding into the spelter socked installed at the top mounting is prepared outside the testing device (see Figure 1c), the testing device has an integrated specimen heating on the bottom mounting to reduce the preparatory effort and possible errors as misalignment and pre-damaging of the struts before testing. For the three-point microbending experiments, no further fixation of the strut is needed. The extracted strut is supported by the two fine support blades on the bottom side of the specimen. A third fine blade acts as bending mandrel in the middle of the strut axis between the two support blades.

In both configurations, microtensile setup and microbending setup, the bottom mounting acts as movable part for load application. While the upper spelter socket is fixed in position, the lower socket is moved down to apply tensile loading and it is moved up to realise a bending of the strut by the bending mandrel.

### 2.3. Geometrical Reconstruction with Photogrammetry

The complex and individual geometry of each strut complicates the stress calculation which is required if material parameters have to be computed from the force-deflection curves. To facilitate the calculation, the exact geometry of each strut is needed. Here, photogrammetry is used as cost-efficient alternative to X-ray computed tomography. A custom-built setup is used for photogrammetric reconstruction of a surface model of each strut. The extracted struts are aligned on the centre of a defined pattern mounted on a rotating table. This setup rotates the strut specimen in 24 steps around its longitudinal axis. During this process, images are taken in two different translation angles. More details on the custom-build setup and the used photogrammetric method can be found by Bleistein et al. [19]. The edges of the strut specimen, combined with the shadows produced from the specimen paired with an underlying pattern enable an accurate three dimensional reconstruction of the strut by using the commercial software 3DSOM^®^(DSL Limited, London, UK). A high resolution 9 MPx CCD camera (Manta G917B, Allied Vision Technologies GmbH, Stadtroda, Germany) with a precise lens (LM50HC, Kowa Optimed GmbH, Duesseldorf, Germany) and a 20 mm spacer reaching 5.5 µm pixel size guarantee for a high precision of the reconstruction.

### 2.4. High Precision Digital Image Correlation

The small dimensions of the struts require a high resolution of the optical measurement system and hence a very fine speckle pattern for the analysis of local strains by DIC. For the optical analysis of the deformation, a 9 MPx CCD camera (Manta G917B, Allied Vision Technologies GmbH, Stadtroda, Germany) with a bi-telecentric lens (DTCM110-16.6, VICO Technology CO., LTD, Shenzhen, China) reaching a pixel size of 3.69 µm is used. To reduce scattering during the DIC evaluation the black speckles of the speckle pattern should consist of three to five pixels. In the case of a thought experiment with ideally round speckles, the diameter of each speckle would be between 6 µm and 10 µm and covering 30% of the surface area with black speckles [20]. This speckle size and speckle density guarantee the best measurement quality for the combination of specimen, camera and lens.

The speckle density is determined using histograms of the gray levels of 8 bit monochrome images taken from the CCD camera. In these images, each pixel has a value between 0 and 255, where 0 is black and 255 denotes to white. All pixels with a value unequal to 0 or 255 are gray. For a good DIC correlation the background should contain pixels between 240 and 255, which means that around 70% of the pixel should be white or slightly gray. The pixels of the speckles should have a gray value from 0 to 45 depending on the point’s shape and the location of the pixels. An ideal black for all pixels forming a speckle is not attainable. Nevertheless, a good pattern can be identified by a histogram of gray levels with one maximum around 25, a second one around 247 and almost no levels in between this range.

**Figure 2:**
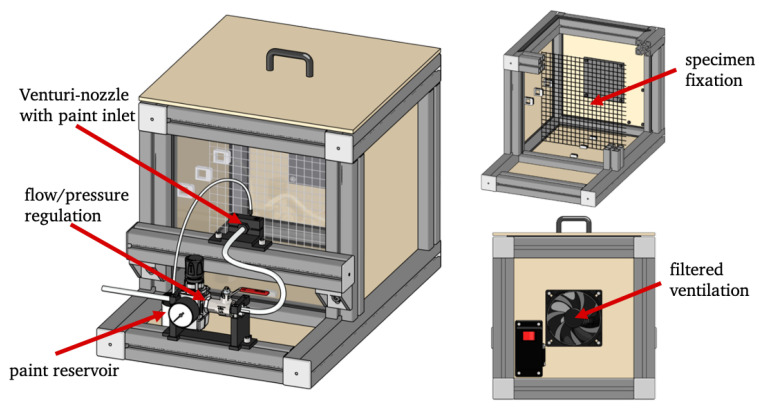
Setup to produce perfect speckle pattern for each individual DIC application.

The conventional method to produce the speckle pattern is spray painting with a spray can or an airbrush pistol. Therefore, the quality of the speckle pattern is strongly user-dependent. In a manual process, small equally sized and well distributed speckles are almost unreachable. To remove the user dependency of this process and to guarantee for a high quality of the speckle pattern on each strut specimen, a custom-made speckle setup is developed (see Figure 2).

The three main parts of the speckle setup are a 3D printed Venturi-nozzle, different-sized paint inlets and a pressure regulator. The Venturi-nozzle allows for the paint intake out of the paint inlet based on Bernoulli’s principle. Different-sized cannulae are use as variable paint inlets to change the amount of paint nebulised by the compressed air coming from the pressure regulator. The paint used in this work is a mixture of a black airbrush primer (Surface Primer Black (Acrylic-PU) from www.vallejo-farben.de) and water in the ratio 1:1. The advantage of the primer compared to normal sprac ink is the lack of clear varnish, which normally leads to reflections and hence errors in DIC evaluation. Variations in the three main parts, Venturi-nozzle, paint inlet and pressure regulator allow finding ideal parameters producing the correct speckle size and density for each specific application. Additionally, the housing of the setup and the filtered air flow ensure that no influence on the user or the environment occurs during the application of the speckle pattern. This custom-made setup reproducible produces equally sized speckles with an optimal size of about five pixels for all experiments and an ideal speckle density of 30% to 40% resulting in an optimal black to white ratio proven by the histograms.

Beside the application for local strain evaluation on single struts of metal foams the custom-build setup allows for speckles in all desired sizes not only for small specimens such as individual struts or pores but also for applying reproducible optimal speckle patterns on specimens up to an edge length of 100 mm.

### 2.5. Investigation of Cross-Sectional Area

Due to the complex surface structure of an open-cell foam, the coating thickness can vary locally. Furthermore, the cross-sectional area for the individual struts of the purchased PU foam is not known. To analyse the size of the PU core and the thickness of the Ni coating, five struts are cut from different positions in a Ni/PU hybrid foam sample. All five struts are glued onto the bottom of the mould, ensuring an orthogonal alignment with respect to the floor. The specimens are embed in Technovit 4002IQ, Kunzler GmbH, Hanau, Germany guaranteeing a fissure-free casting. Along the strut length, 33 micro-sections are prepared. The simultaneous embedding of all five struts enables an equal cutting. Each of these 33 polishes are realised in two grinding steps. In the first step a sandpaper of 600 grain size and in the second step a sandpaper of 1200 grain size is used. The use of sandpaper of two coarsenesses is necessary to get an acceptable milling rate using the coarse paper and a sufficient surface quality using the fine paper. For each micro-section an image is captured using optical microscopy (Axio Imager.M, Carls Zeiss AG, Oberkochen, Germany) with an optical magnification of 20×.

**Figure 3:**
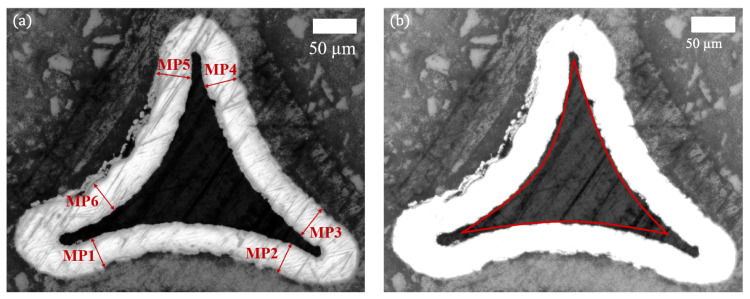
(**a**) micro-section of a strut with highlighted positions of coating thickness measurement (MP1-MP6); (**b**) edited micro-section showing the size estimation of PU core.

For a quantitative comparison of the coating thickness between the different struts, six measuring points (MP1-MP6) were chosen in each image. The selection of these points is based on the triangular shape of the struts, so that the points are distributed more or less equidistantly along the border. To illustrate the positioning, an exemplary micro-section with the highlighted measurement points is displayed in Figure 3a.

The cross-sectional size of the PU core is measured with the software ImageJ [21,22]. Therefore, the brightness of the image is enhanced and the area of the PU core is selected using the *versatile wand tool* plugin. An exemplary image with the enhanced brightness and the PU core is depicted in Figure 3b.

### 2.6. Stress Calculation from Experimental Data

For the stress evaluation, a geometrical model of each strut is required. These models allow for the measurement of the cross-sectional area at the crack position for tensile tests or the calculation of the section modulus at the position of the bending mandrel for the three-point bending tests. In tensile tests on PU struts only very small forces occur and therefore the area of the PU cores should not be taken into account when calculating the stress in hybrid foams with very stiff Ni coatings. In addition, measurements using micro-sections showed the same cross-sectional area for all five PU cores. Therefore, a constant core can be subtracted from the geometry of the struts to calculate the stress for the tensile tests and the section modulus for the bending tests for the coated struts. This results in geometric models of hollow struts that represent the pure nickel coating and which are responsible for the resulting stress and the section modulus of the coating.

To estimate the crack position in the tensile tests, the distance between the lower moulding and the crack is measured as a percentage of the strut length. Based on the geometrical model, a stack of images is generated using the open source software ImageJ [21,22]. In this image stack, the 11 images around the crack are extracted. The average size of the PU core is subtracted from the mean cross-sectional area in these 11 images. The resulting area is used for stress calculation.

In comparison to tensile tests, the stress during bending is not constant over the entire cross-sectional area of the strut. The bending stress depends on the distance to the neutral axis and the distance to the loading. The position of the neutral axis is defined by the geometry of the cross-sectional area. The magnitude of the bending stress $\sigma_{b}$ in tensile direction is maximum on the bottom side of the strut and in the compression direction is maximum at the loading side of the strut. For the calculation of the maximum stress, it is possible to divide the bending moment $M_{b}$ by the section modulus $Z_{p}$

(1) $$\sigma_{b}\;=\;\frac{M_{b}}{Z_{p}}.$$

For three-point bending the bending moment $M_{b}$ is defined as

(2) $$M_{b}\;=\;\frac{F\;l}{4},$$

with the applied force *F* and the distance between the supports being *l*. The section modulus $Z_{p}$ is determined using the *BoneJ2* plugin in ImageJ. For the calculation of the section modulus, a geometrical surface model of the strut is sliced into an image stack and the geometry of 11 images near to the bending mandrel is chosen. A geometric model of a constant core is subtracted from this strut geometry to produce a geometric model of the Ni-coating. The *BoneJ2* plugin allows the rotation of the coordinate system to the coordinate system of the experiment and then calculates the section modulus as a mean over these slices of the Ni coating. Thus, the real cross-sectional shape of the struts is reconstructed from the images and no simplification of the geometry is necessary for the evaluation of the bending stress. Due to the use of Euler-Bernoulli beam theory, the resulting bending stress is only valid for the elastic part of the deformation up to the yield point.

**Figure 4:**
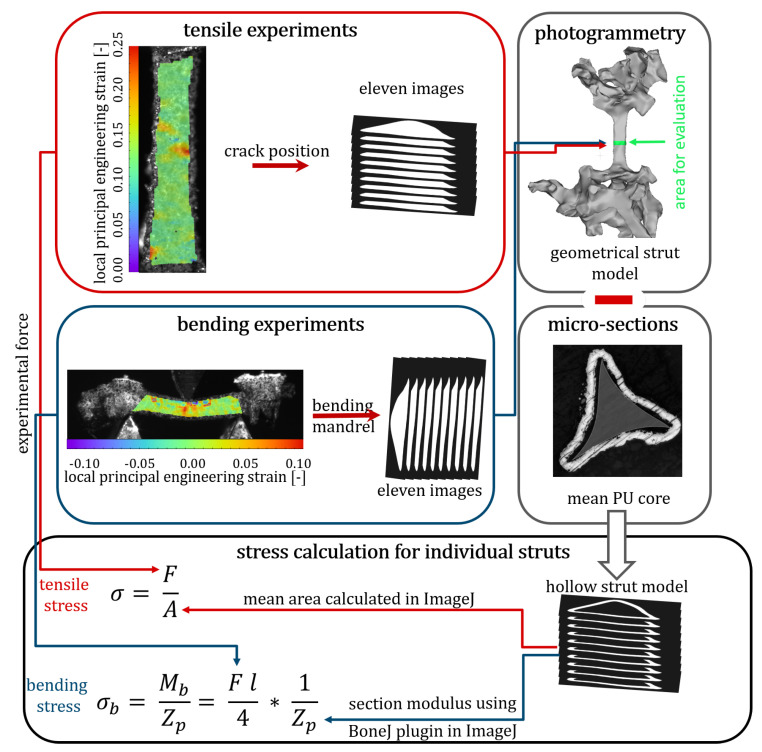
Overview of evaluation steps for stress calculation in individual Ni/PU hybrid foam struts during microtensile and microbending experiments.

Figure 4 visually summarises the entire process of experimental testing and evaluation of the stress calculation described above.

## 3. Results

### 3.1. Investigation of Cross-Sectional Area

**Figure 5:**
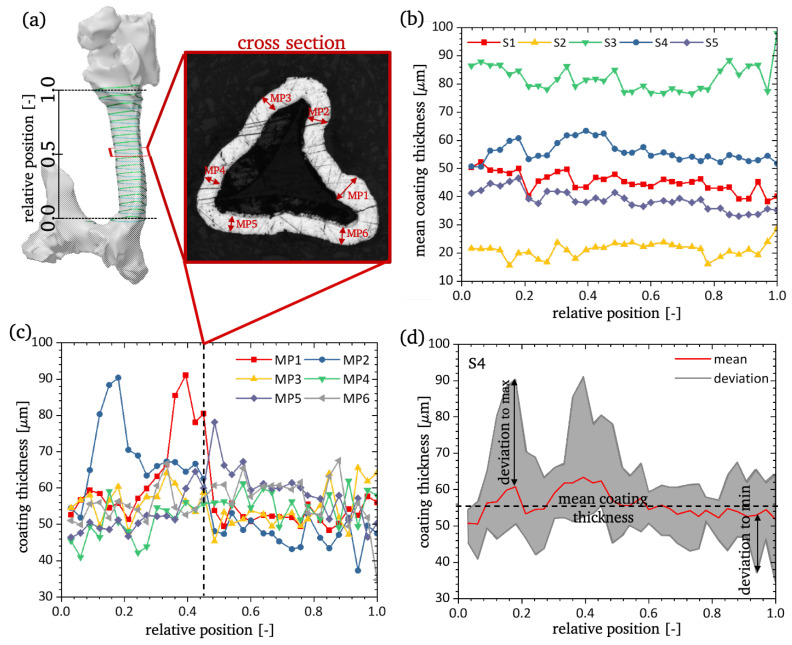
(**a**) Scheme of micro-sections for strut S4; (**b**) mean coating thickness for all five struts (S1–S5); (**c**) coating thickness at six measuring points (MP1–MP6) for one strut (S4); (**d**) mean coating thickness and scatter band for one strut (S4) with marked position of maximum and minimum deviations.

All struts were extracted from the same macroscopic Ni/PU hybrid foam sample; however, the scattering in coating thickness is large. The coating thickness is measured in 33 micro sections along the struts length and six measuring points per micro-section are analysed as described in Section 2.5. The thinnest mean coating thickness for a strut is 21.26 μm, whereas the thickest mean coating thickness is 82.13 μm. The average coating thickness for the five different struts (S1–S5) can be found in Figure 5b. The mean coating thickness of three struts (S1, S4, S5) are close to the targeted thickness of 40 μm. Hence, the coating thickness is constant over the length of the struts and its variation in each micro-section of a strut at different measurement points is minimal.

Figure 5c presents the measured values of the coating thickness at the six measuring points (MP1-MP6) for all 33 slices of an exemplary strut. Therefore, single values show huge deviations from the mean coating thickness. For the strut S4 the maximum value is 91.11 μm, which equals a deviation of 29.60 μm (48.12 %) from the mean value in this micro-section (see Figure 5d). This local increase of the coating thickness is produced by the geometry of the cross-section of the struts of the template PU foam. These struts of the template foam show a concave triangular cross-section (see Figure 5a). Based on this geometry, the corners of the triangle are very sharp and local current peaks can appear during electrodeposition, producing high local deposition rates. Despite these local problems, the coating is constant and the scatter of the coating distribution over the length of the strut is small. Moreover, the deviation to thinner layers is also high and cannot be explained as simply as the thicker coating spots and require an adjustment of the process parameters of the electrodeposition process. The mean value of the coating thickness and the corresponding values of the deviation to the minimum and to the maximum value for all investigated struts are outlined in Table 1. Therefore, all struts show a variance from the mean value to the maximum value of 40.81–50.23% and from the mean to the minimum value of 24.69–43.35%.

In addition to the coating thickness, the cross-sectional area of the PU core was measured. The results are displayed in Table 2. In contrast to the coating thickness, the PU core shows a very small variance among the individual struts. The mean cross-sectional size for all five struts is 23,329.06 ± 2494.85 μm${}^{2}$, whereas the minimum mean cross-sectional size found is 20,705.73 μm${}^{2}$ (S2) and the maximum mean cross-sectional size is 26,034.89 μm${}^{2}$ (S4). The difference between the values equals a deviation of 11.24% from the mean to the minimum and 11.60% from the mean to the maximum. Furthermore, the cross-sectional area of the PU core is constant over the length of the strut. Obviously, the micro-sections containing the node area are excluded. These measurement results show that the PU core has no influence on the outer diameter of the individual Ni/PU hybrid foam strut. Additionally, photogrammetric surface models enable the estimation of the coating thickness based on the outer dimensions of each strut. A solid model of the struts, which ignores the inner structure and coating thickness of the strut is used to estimate homogenised material parameters and does not amplify the errors.

### 3.2. Tensile Tests on Individual Struts

**Figure 6:**
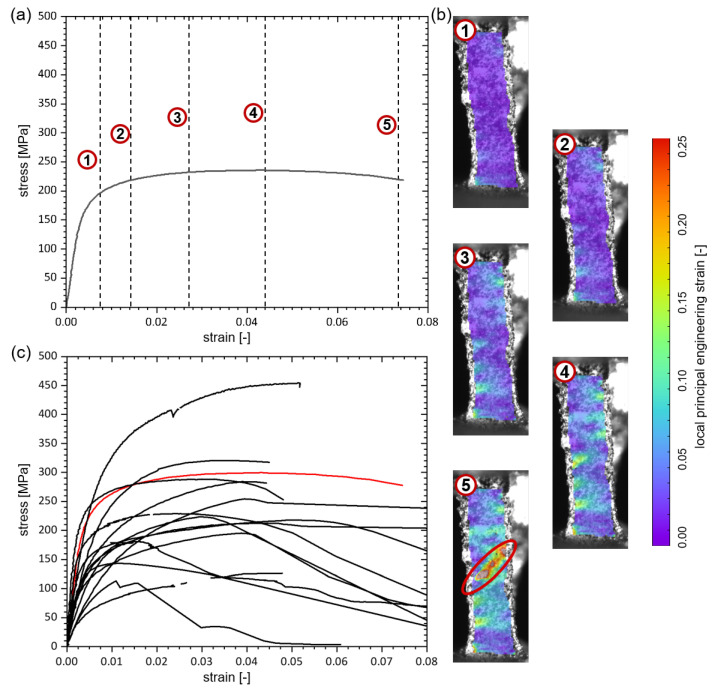
Results of 18 microtensile tests on Ni/PU hybrid foam struts: (**a**) exemplary stress-strain-diagram with five marked positions, (**b**) DIC results for the five marked positions in (**a**) with the crack highlighted (red ellipse) in the last step, (**c**) stress-strain-diagrams for all 18 struts, with the exemplary strut highlighted in red.

Figure 6 (top) outlines the stress-strain-diagrams of 18 struts studied under microtensile loading. In Figure 6 (bottom), a DIC measurement on an exemplary strut for five load steps of the microtensile experiment is shown. DIC is applied to determine to two different values in case of the tensile tests. The first is the strain measurement. For this purpose, three lines were drawn from the upper attachment to the lower attachment and the strain of the lines was evaluated. The mean value of all three lines was then used as global strain. The second is to locate the crack position on the strut axis. The cross-sectional area of the strut in this exact crack position enables the accurate stress calculation. For the estimation of the cross-sectional area, the surface model is generated with the photogrammetric method described in Section 2.3 and evaluated for its cross-section close to the position where the crack will appear as described in Section 2.6. The stiffness under tension was measured as secant modulus in the elastic regime and ranges from 12 GPa to 98 GPa. The calculated stiffness under tension is a parameter resulting from the theoretical cross-sectional area assuming the same PU core for all tested struts. The struts are much lesser stiff compared to the Young’s modulus of solid Ni ($E_{Ni}$ = 207 GPa [23]) due to the local variations in the coating thickness seen in the micro-sections (see Section 2.5).

The strain measurements in DIC on the struts show the appearance of strain localisation bands. The generated strain is not homogeneously distributed over the complete sample. Some areas stay almost undeformed and the total strain is seen to be localised to a few areas. The cross-sectional areas show almost no necking and the crack is brittle. This brittle failure results from the nanocrystalline grain structure of the Ni coating [24,25]. Additional to the Ni coating the foam consists of the PU template and its influence on the hybrid material response needs to be determined. For all struts, the PU core is still intact after fracture of the Ni coating and shows a large strain. After the cracking of the Ni coating, the measured force in all tests is below 0.2 N and with that below the resolution of the used force sensor. Nevertheless, this is an important observation for the modelling of Ni/PU hybrid foams, in contrast to Al foams where after failure no bonding exist.

### 3.3. Three-Point Bending Tests on Individual Struts

**Figure 7:**
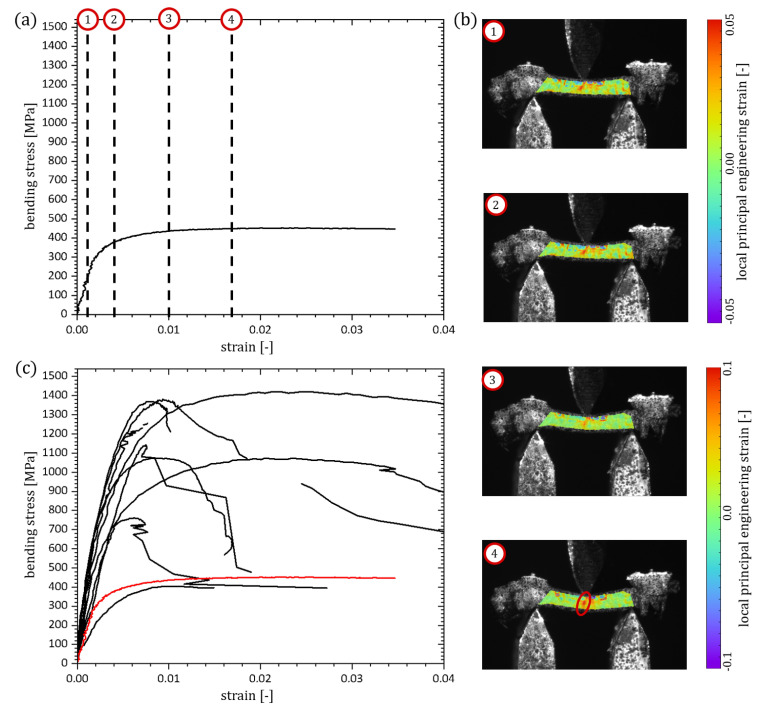
Results of 10 bending tests on Ni/PU hybrid foam struts: (**a**) exemplary bending stress-strain-diagram with four marked positions, (**b**) DIC results for the four marked positions in (**a**), with the crack highlighted (red ellipse) in the last step, (**c**) stress-strain-diagrams for all 10 struts, with the exemplary strut highlighted in red.

The stress-strain-diagrams of the microbending tests on the Ni/PU struts are shown in Figure 7 (top). The diagrams show a good correlation in the first part of the curves until the yield points are reached. Here, the method used for the calculation of the maximum bending stress is only valid before local yielding occurs. Hence, only the elastic part of the curves up to the yield point can be used for the comparison with each other. During the bending tests on individual struts of a Ni/PU hybrid foam a similar behaviour for all struts was visible in the DIC measurements. After reaching the yield point, a sharp increase of the strain occurs locally at different positions in the specimens. An exemplary DIC measurement and the corresponding stress-strain-diagram (red line) can be found in Figure 7 for one strut at the highlighted positions ① to ④.

In elastic beam theory a distribution is propagated from positive strain at the far side to negative strain on the loading side with a strain of zero at the neutral axis. The displayed DIC measurement cannot confirm this statement for bending tests on individual struts since there is not only elastic loading of the struts but also plasticity as seen in the stress-strain-diagrams. In contrast, the measurement shows localised strains and a progression of these areas from the opposite side of the force to the force loading side. The strain increases during loading until the Ni coating cracks at one of these points either in the area of maximum compression or maximum tension.

Similar to the tensile tests the PU core remains intact after fracture of the Ni coating and still connects the two parts of the strut. The reaction force produced from the remaining PU is very low. It is under the resolution of the force sensor and cannot be precisely measured.

Based on all these observations, only the elastic part of the stress-strain-diagrams can be evaluated corresponding to the loading states ① or ② in Figure 7. The stiffness under bending was determined according to the stiffness under tension as secant modulus in the elastic regime of the bending stress-strain diagrams. Hence, it should be directly comparable to the stiffness under tension and hence to the Young’s modulus but it is not equal to the so-called bending stiffness, which is the product of Young’s modulus an area moment of inertia. The stiffness under bending of the individual struts differs between 144 GPa and 482 GPa, which equals a difference of 70.1%. Reasons for these variations can be found in the manufacturing process and geometry of the struts [8,9]. The coating produces a nanocrystalline Ni layer on top of a graphite lacquer which is applied on the PU template during the coating process of the hybrid foam. This process is not free of errors and small defects in form of cavities or inclusions can appear. Using the real section modulus of the struts as calculated in Section 2.6 excludes only the coating thickness as an influencing parameter and not any defects in the coating itself. As in the tensile tests, these errors lead to a scattering of the experimental results. Here, the position of the defect can cause a further amplification of the error. The imhomogeneous distribution of stresses during bending will result in a huge decrease of the effective yield point of the strut, if a cavity exists at the position of maximal bending stress. Performing real in situ three-point bending tests on single struts is very time-consuming and needs very specialised equipment. Furthermore, FE-simulations with full resolution of all defects is too intensive in terms of time and capacity to perform on a larger number of struts. The large scattering in the material properties clearly outlines the problem in simulation of open-cell foams and their struts. In contrast to macroscopic specimens cut from bulk material, it is not possible to determine one single material parameter for individual struts due to the large variation in microdefects. Hence, a homogenisation of the material properties of Ni/PU foam struts accounting for all microdefects in a statistical sense is necessary for an effective simulation of Ni/PU hybrid foams.

## 4. Discussion

Bending and buckling are the main deformation modes on strut level in open-cell metal foams. Since the deformation of individual struts strongly affects the macroscopic material behaviour of entire foams, it is of paramount importance to study the material behaviour of individual struts. Nevertheless, performing experiments on individual struts is a very challenging task due to the very complex boundary conditions and load application resulting from the small sample dimensions and the non-standardised and individual sample geometry. Hence, only a few studies in the literature deal with strut testing. Unfortunately, only two publications deal with microbending experiments on the micro scale of metal foam. Only one of these focusses on struts of open-cell metal foam. So far, however, Reis et al. [15] have only provided the basis for the evaluation of microbending tests on individual foam struts by developing a method for high-precision DIC measurements on metal foam struts. An actual determination of material parameters from bending tests did not take place so far. Hence, the current study is the first work dealing with microbending experiments on individual open-cell metal foam struts and the development of an evaluation routine to determine material parameters from such experiments. The presented experimental method and the evaluation routine allow for the determination of the bending stiffness of individual foams struts as it is acting in macroscopic foams under deformation.

The difference between the mean stiffness under tensile loading and the mean stiffness under bending for the three-point bending tests is around 187 GPa. Ideally, both should be identical and should also be identical with the Young’s modulus. For bending, the material is up to five times as stiff as during tensile loading in the elastic region. Even the weakest tested struts show a higher stiffness under bending than the stiffest struts under tensile loading, which is not only an effect of the material itself but also depending on the geometry of the struts as well as idealisations during the evaluation. The resistance against tensile loading is only influenced by the cross-sectional area. The specific geometry of this area is not relevant. In contrast to that, the resistance against bending is dominated by the real specific geometry and outer dimensions of the cross-section resulting in a specific area moment of inertia. This made coated struts with a soft PU core and a very hard outer Ni layer to be the perfect structure for multi-axial load cases and justifies the main use of the material as crash absorber, where the macroscopic foam is compressed but the majority of the struts are bent.

During the testing of foams on the microscale, the geometry of the struts and the heterogeneous microstructure have a large influence on the resulting stress-strain-diagrams and lead to a scattering of the results [26]. For an Al alloy foam Amani et al. [27] were able to proof that a modelling routine using microstructure- and structure sensitive models can predict the failure behaviour. This required a high-resolution FE-mesh with different material information per element based on microstructure information from computed tomography of the struts. The effort required for this is clearly too high to be carried out for a large number of experiments. Nevertheless, the results of the work of Amani et al. [27] suggest that the variance of the coating thickness and the geometry of the Ni/PU hybrid foams investigated here lead to a large scattering of the measurement results. The aim should therefore not be to calculate each individual strut at full resolution, taking into account all defects, but rather to determine stochastic material parameters that can map the macroscopic behaviour of the Ni/PU hybrid foam. To identify these stochastic parameters has to be the next step of the current work.

To improve the accuracy of the experimental results, further experiments should be performed changing the setup from tree-point bending to two-point bending to overcome problems with the alignment of the struts in the three-point bending setup. Due to the strongly pronounced structure-property relationship of metal foams, the material parameters of the struts determined in this way are of decisive importance for predicting the macroscopic behaviour of metal foams by finite element simulations. Future work will focus on the development of a virtual lap for the design of components made of Ni/PU hybrid foams by a stochastic material model using the material parameters determined from microtensile and microbending experiments including the scattering in the material parameters and accounting for individual defects as shown experimental results.

## 5. Conclusions

The coating of a cost-efficient material such as PU foams with a high performance material such as a Ni coating allows not only to change the material behaviour of the new hybrid material significantly but also to tailor the properties application-specifically by varying the coating thickness. The mechanical properties of the Ni/PU hybrid foam are dominated by the stiffness of the Ni coating. Unfortunately, the electrochemical coating process is not perfect due to mass transport limitations resulting in inhomogeneities in the coating thickness.

The present study dealt with the determination of micromechanical material properties from individual struts of Ni/PU hybrid foams by microtensile and microbending experiments. While tensile tests can already be found in the literature, it is the first time that microbending tests on individual struts of metal foams were performed and evaluated for the determination of micromaterial parameters. Due to non-homogeneous coating, the distribution along and around several Ni/PU hybrid foam struts was analysed by 33 micro-sections perpendicular to the strut’s longitudinal axis. The coating thickness is constant for each individual strut over its length but it depends on the strut’s position in the foam. Since the cross-sectional area of the struts of the PU core was found to be constant, the Ni coating thickness can be estimated from the outer dimensions of the strut only. This was used for stress evaluation. The large scattering in the stress-strain diagrams of microtensile and microbending experiments in combination with the local strain bands measured by DIC indicate that local defects in the struts are responsible for local softening caused by this microdefects. Regarding elastic deformations and hence comparing the stiffness calculated from tensile tests and bending tests, respectively, the stiffness under bending shows a lower scattering. In both types of experiments the PU core remains intact after the failure of the Ni layer. Such micromechanical experiments on individual struts for the determination of micromechanical material parameters are of paramount importance for the simulation of the macroscopic material behaviour of metal foams by finite element simulations and hence for the application-specific optimisation of metal foam components.

## Figures and Tables

**Table 1:** Coating thickness of five struts (S1–S5) measured in 33 micro-sections along the length of a strut and along six measuring points (MP) per micro-section.

	S1	S2	S3	S4	S5
mean (μm)	21.16	45.33	82.13	55.93	38.86
max (μm)	43.31	67.93	119.78	91.11	65.67
min (μm)	6.97	26.98	48.17	37.28	25.23
deviation to maximum (μm)	14.48	19.69	37.89	29.60	19.11
deviation to maximum (%)	50.23	40.81	46.47	48.70	48.70
deviation to minimum (μm)	11.82	21.67	28.93	15.67	9.28
deviation to minimum (%)	41.00	43.35	37.13	29.59	24.69

**Table 2:** Cross-sectional area of PU core for five struts (S1–S5).

	S1	S2	S3	S4	S5
mean (µm${}^{2}$)	20,789.26	23,251.76	24,225.03	25,994.73	20,673.55
max (µm${}^{2}$)	23,237.60	26,133.05	27,825.85	27,927.93	22,357.37
min (µm${}^{2}$)	17,863.06	21,181.21	21,082.99	23,140.64	16,866.58
deviation to maximum (%)	11.78	13.17	14.82	7.27	7.98
deviation to minimum (%)	14.08	8.27	12.80	11.12	18.54

## Abbreviations

The following abbreviations are used in this manuscript:

Al	aluminium
CCD	charge-coupled device
DIC	digital image correltion
*F*	force
*l*	length
$M_{b}$	bending Moment
MP	measuring point
MPx	megapixel
Ni	nickel
PCS	plastic collapse stress
ppi	pores per inch
PU	polyurethane
S	strut
X-ray	high-energy electromagnetic radiation
$Z_{p}$	section modulus
2D	two dimensional
3D	three dimensional
$\sigma_{b}$	bending stress
